# A considerable fraction of soil-respired CO_2_ is not emitted directly to the atmosphere

**DOI:** 10.1038/s41598-018-29803-x

**Published:** 2018-09-10

**Authors:** Enrique P. Sánchez-Cañete, Greg A. Barron-Gafford, Jon Chorover

**Affiliations:** 1 B2 Earthscience, Biosphere 2, University of Arizona, Tucson, 85721 USA; 2 Departamento de Física Aplicada, Universidad de Granada, Granada, 18071 Spain; 3 School of Geography and Development, University of Arizona, Tucson, 85721 USA; 4 Department of Soil, Water and Environmental Science, University of Arizona, Tucson, 85721 USA; 5 IISTA-CEAMA, Instituto Interuniversitario de Investigación del Sistema Tierra en Andalucía, Granada, 18006 Spain

## Abstract

Soil CO_2_ efflux (*F*_*soil*_) is commonly considered equal to soil CO_2_ production (*R*_*soil*_), and both terms are used interchangeably. However, a non-negligible fraction of *R*_*soil*_ can be consumed in the subsurface due to a host of disparate, yet simultaneous processes. The ratio between CO_2_ efflux/O_2_ influx, known as the apparent respiratory quotient (ARQ), enables new insights into CO_2_ losses from *R*_*soil*_ not previously captured by *F*_*soil*_. We present the first study using continuous ARQ estimates to evaluate annual CO_2_ losses of carbon produced from *R*_*soil*_. We found that up to 1/3 of *R*_*soil*_ was emitted directly to the atmosphere, whereas 2/3 of *R*_*soil*_ was removed by subsurface processes. These subsurface losses are attributable to dissolution in water, biological activities and chemical reactions. Having better estimates of *R*_*soil*_ is key to understanding the true influence of ecosystem production on *R*_*soil*_, as well as the role of soil CO_2_ production in other connected processes within the critical zone.

## Introduction

Soil carbon dioxide (CO_2_) efflux is the second largest contributor to terrestrial CO_2_ exchanges, similar in scale to uptake by terrestrial photosynthesis^1,2^. Soil CO_2_ efflux (*F*_*soil*_) is defined as the rate of CO_2_ exchange between soil and atmosphere, and it is the result of soil CO_2_ production (*R*_*soil*_) and its transport to the atmosphere. Rates of *R*_*soil*_ are the result of heterotrophic respiration during the decomposition of organic matter by microbes and autotrophic respiration by roots^3^. Both *F*_*soil*_ and *R*_*soil*_ act together in response to the interactions between biotic and abiotic factors^4–6^. Generally, *F*_*soil*_ increases with the productivity of an ecosystem^7^, driven by increases in temperature and precipitation^1,8^. With ample water, temperature is the dominant driver of *F*_*soil*_, however, in arid and semiarid ecosystems, patterns of *F*_*soil*_ are often driven by precipitation pulses^9–13^ and variation in soil moisture.

*F*_*soil*_ can be measured using manual or automatic chambers^14,15^ that capture CO_2_ emitted from the soil surface to the atmosphere or estimated by the gradient method through measures of the soil CO_2_ molar fraction at multiple depths^16,17^. Commonly, *F*_*soil*_ is considered equal to *R*_*soil*_, and the two terms are used interchangeably within the literature and in land surface models. However, a considerable fraction of the *R*_*soil*_ can fail to actually emerge from the soil surface (*F*_*soil*_) due to a host of different processes, such as aqueous phase partitioning^18^, calcite dissolution reactions^19^, gravitational percolation due to a higher density^20^, or CO_2_ dissolution in xylem water^21^. Therefore, simple estimations of *F*_*soil*_ are likely lower than actual rates of *R*_*soil*_. Misrepresenting *F*_*soil*_ as *R*_*soil*_ can have significant consequences for interpretation of both biotic and abiotic processes because it not only underestimates the contributions of aboveground function to belowground processes, but it also yields a misguided understanding of the rates and drivers of subsurface biogeochemistry and the potential for carbon exports from the system through hydrological transport.

The importance of these alternative CO_2_ loss pathways is illustrated when considering that soil can store an order of magnitude greater CO_2_ as dissolved inorganic carbon (DIC, inclusive of dissolved CO_2_, carbonic acid, bicarbonate, and carbonate) in the aqueous-filled relative to gas-filled pore space^22^. As a result, large CO_2_ losses can be produced by DIC leaching in all ecosystems around the world, with increased CO_2_ losses in ecosystems with higher precipitation and higher soil solution pH. In semiarid regions, this DIC leaching may explain a portion of the missing terrestrial carbon sink^23^. For this reason, distributed measures of O_2_, which has an aqueous solubility 29.7 times lower than CO_2_ at 15 °C and does not form additional chemical species by dissolution in water, provides a useful constraint on determining soil CO_2_ production that might otherwise be missing from *R*_*soil*_.

**Figure 1:**
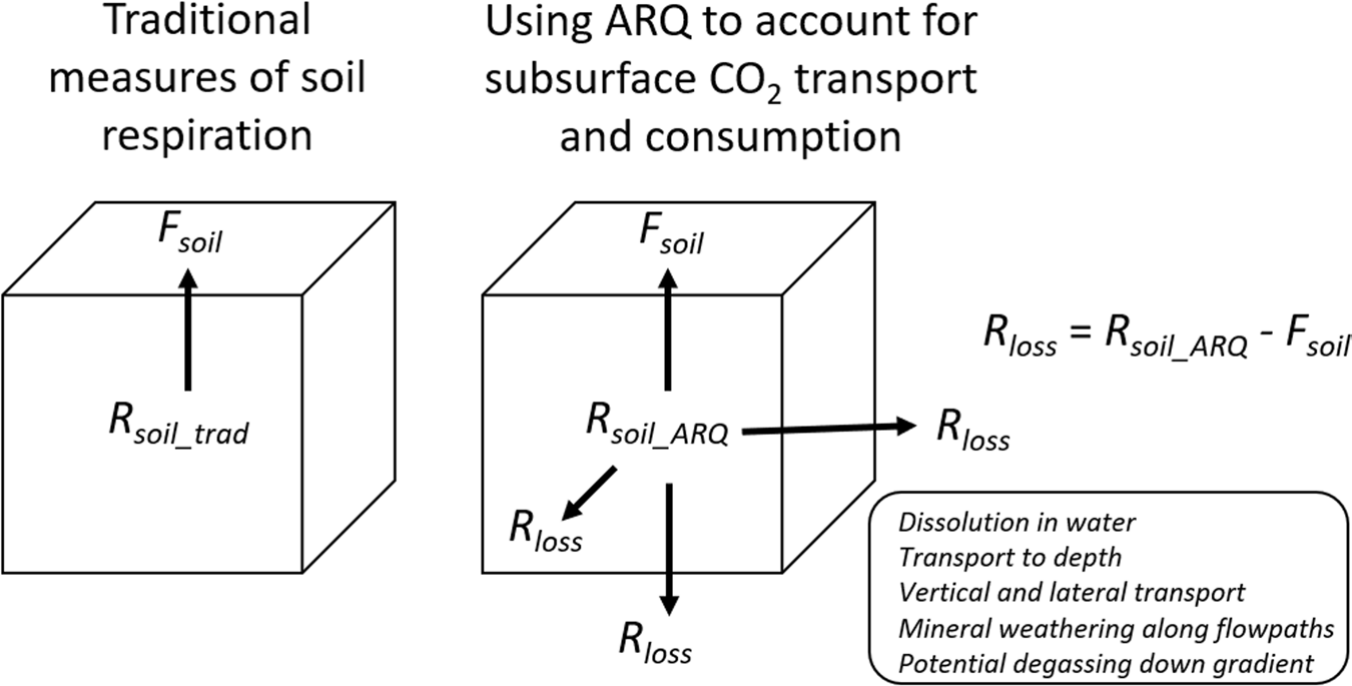
Measurements of *apparent respiratory quotient* (ARQ), i.e., the ratio of soil CO_2_ efflux/O_2_ influx, have the potential to provide improved quantification of soil respiration, and partitioning of soil respiratory CO_2_ into vertical (upward) gaseous and lateral or downward dissolved fluxes. Here, *R*_*soil*_ is the soil CO_2_ production measured, either using the traditional efflux method (*R*_*soil_trad*_) or on the basis of ARQ (*R*_*soil_ARQ*_). *F*_*soil*_, which is the soil CO_2_ (upward) efflux estimated by the CO_2_ gradient method, is typically equated to soil respiration (*R*_*soil_trad*_). However, direct continuous measurements of ARQ, as conducted in the current work, reveal that a significant fraction of CO_2_ produced by soil respiration is transported or consumed in the subsurface, and not locally emitted to the atmosphere. Hence, a substantial amount of respired CO_2_ – unaccounted for by quantifying *F*_*soil*_ alone, and denoted here as *R*_*loss*_ *–* can be estimated on the basis of concurrent measures of O_2_ influx to soil. The ARQ method reveals a significant component of soil respiration (*R*_*loss*_) that is not emitted locally to the atmosphere. *R*_*loss*_ is the CO_2_ produced, but unaccounted for, in traditional measures of CO_2_ surface efflux. This *R*_*loss*_ is consumed by subsurface processes attributable to dissolution in water, vertical and lateral transport along hydrologic flow paths, chemical reactions (including, e.g., silicate and carbonate mineral weathering), and potential degassing upon groundwater discharge (e.g., to streams). Non-negligible values of *R*_*loss*_ indicate that (i) flux based measurements alone significantly underestimate local soil respiration and (ii) an important fraction of soil respiratory CO_2_ production is consumed in subsurface reactions. *R*_*soil_ARQ*_ is the total soil CO_2_ production, and the sum of *R*_*soil*_ and *R*_*loss*_.

The ratio of soil CO_2_ efflux to O_2_ influx, known as the *apparent respiratory quotient* (ARQ), allows one to estimate the CO_2_ losses from *R*_*soil*_^22^. A diagram, with the main variables involved in exchange and loss of CO_2_, is shown in Fig. 1. Here we present the first study using continuous ARQ estimates to evaluate annual CO_2_ losses of carbon from *R*_*soil*_ (*R*_*soil_ARQ*_, where *R*_*soil_ARQ*_ = *R*_*soil*_ + *R*_*loss*_). Our goals were (*i*) to quantify the values, patterns, and seasonality of ARQ at different soil depths within a semi-arid coniferous forest and then (*ii*) to estimate the amount of soil CO_2_ removed through biological and non-biological processes (*R*_*soil_ARQ*_) (*iii*) in order to illustrate the disparity between *F*_*soil*_ using traditional assumptions that *R*_*soil*_ = *F*_*soil*_ and an estimate of *F*_*soil*_ that takes into account CO_2_ losses (*R*_*loss*_) and actual rates of *R*_*soil*,_ as determined using the ARQ. Having better estimates of *R*_*soil*_ is key to understanding the true influence of aboveground production on *R*_*soil*_, CO_2_-induced mineral weathering, and other biologically-driven processes within the critical zone.

## Results

**Figure 2:**
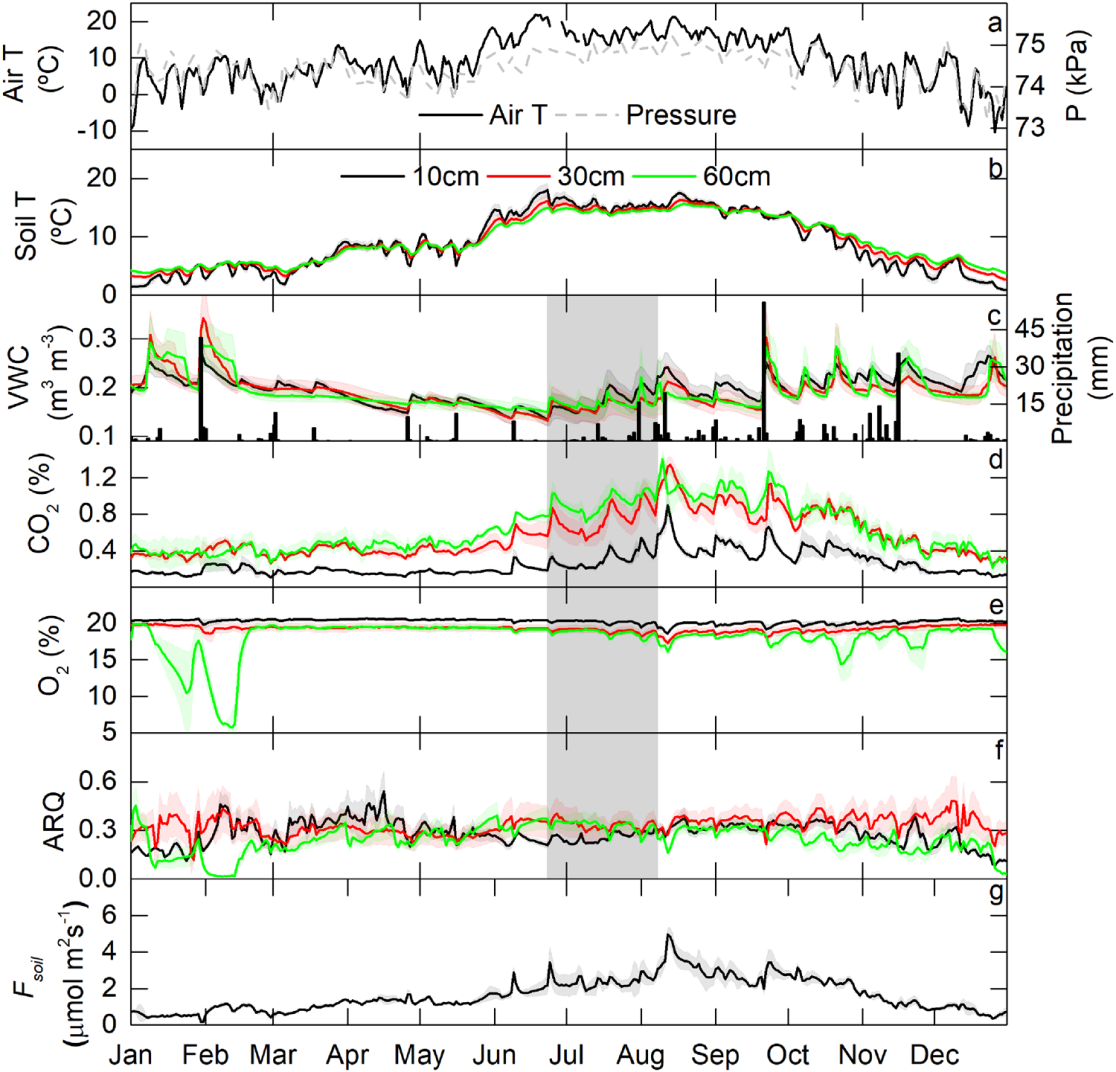
Time series of daily-averaged values for the three pedons of air temperature (Air T), atmospheric pressure (P), soil temperature (Soil T), volumetric water content (VWC), precipitation, CO_2_ volumetric fraction, oxygen volumetric fraction, apparent respiratory quotient (ARQ) and soil CO_2_ efflux (*F*_*soil*_) at 10, 30, and 60 cm depth during 2015. The standard error for each variable is shown with shading. The period studied in Fig. 2 is highlighted with shading.

The annual time series of climatic and edaphic variables are shown in Fig. 2. During 2015, mean air temperature was 9.4 °C, ranging from −10 to 22 °C with synoptic scale fluctuations driven by atmospheric pressure variations associated with passing of frontal systems (Fig. 2a). Mean soil temperature across all depths was *ca*. 9.3 °C, with variability decreasing in amplitude with depth (Fig. 2b). Volumetric soil water content (VWC) averaged 20% across all depths with variation over time driven by rainfall events, falling mainly during the monsoon period (typically July-October; Fig. 2c). In 2015, however, the precipitation period extended until mid-November due to an *El Niño* southern oscillation event. The high VWC measured in January-February was due to snowmelt. When precipitation intensity was greater than 3 mm in 30 min, the delay between precipitation and a VWC response was less than 30 min.

Dynamics of the variables considered to control soil gas concentrations and their exchange with the atmosphere are shown in Fig. 2d–g. Mean CO_2_ volumetric fraction increased with depth, with average values of 0.25, 0.57 and 0.64% at 10, 30 and 60 cm, respectively. We found a clear annual pattern analogous to the temperature pattern, with maxima in summer and minima in winter. Superimposed on this seasonal trend is pulsed increases in the volumetric fraction of CO_2_ driven by precipitation events, with larger amplitude responses during warmer months. Mean O_2_ volumetric fraction decreased with increasing depth from 20.27%, to 19.27% and 18.04% at 10, 30 and 60 cm, respectively. The mean O_2_ volumetric fraction was significantly different at the three depths, and this difference was sustained through the entire year (F_2,336_ = 213.9; P < 0.05). Minimum O_2_ values occurred in the deepest depths during the snowmelt period, and O_2_ variations were anti-correlated with CO_2_ at 10 cm (R^2^ 0.94, p > 0.05) and 30 cm (R^2^ 0.89, p > 0.05) throughout the year. However, at 60 cm a poor correlation (R^2^ 0.11, p > 0.05) was found due to the decoupling during the snowmelt. When the snowmelt period (from January 8 to February 20) was excluded from regression analysis, the correlation between O_2_ and CO_2_ increased notably in deeper layers, with R^2^ values of 0.95, 0.92 and 0.46 at 10, 30 and 60 cm, respectively. Large O_2_ fluctuations at 60 cm during the snowmelt period could be due to the snowmelt during daytime producing a wetting front that percolates to lower permeability soil horizons (higher clay content) at depth, stimulation of soil respiration and hence O_2_ consumption, but with near saturation conditions limiting diffusion of O_2_ into the soil from above. ARQ showed similar mean values at all depths (*ca*. 0.3), reaching minimum values at 60 cm during snowmelt (January-February) and maximum values at 10 cm in April. *F*_*soil*_ was at its maximum during summer and minimum during winter, with an annual mean of 1.64 µmol m^2^ s^−1^. Means, standard deviations, minima, maxima, and correlation coefficients for variables shown in Fig. 2 are included in *Supplementary Information* (Tables 1S and 2S). Monthly descriptive statistics for edaphic variables and ARQ are also included there (Fig. 1S).

**Figure 3:**
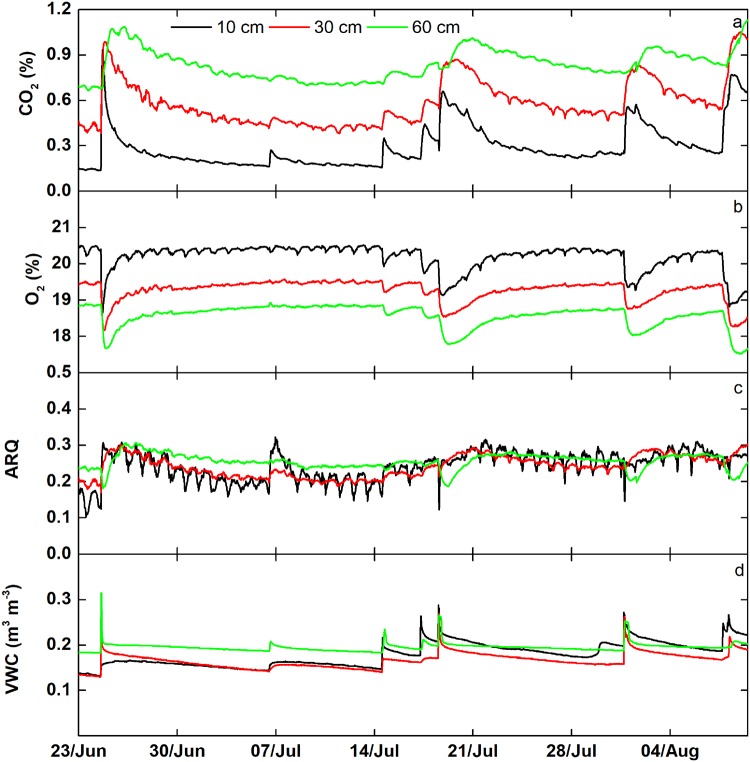
Half-hour averaged values of CO_2_ volumetric fraction, O_2_ volumetric fraction, apparent respiratory quotient (ARQ) and volumetric water content (VWC) at 10, 30 and 60 depth in a single instrumented pedon (north-facing) during the summer monsoon of 2015.

We also examined, in one soil pedon at 30 min averages, the dynamic behaviour of CO_2_ and O_2_ through several rain pulse events to capture their combined effects on ARQ (Fig. 3). ARQ slightly increased at 10 cm and 30 cm in response to rain pulses, but remained stable at 60 cm. Interestingly, the rapid increases in CO_2_ induced by rain events were counteracted by rapid decreases in O_2_, causing only small variations in the ARQ range (c.a. 0.2–0.3). The time to return to values similar to those prior to the precipitation event for CO_2_, O_2_, ARQ and VWC was not delayed with depth. At 10 cm depth, diurnal ARQ fluctuations showed a higher amplitude than at deeper depths, driven by higher amplitude in the O_2_ fluctuations at 10 cm.

**Figure 4:**
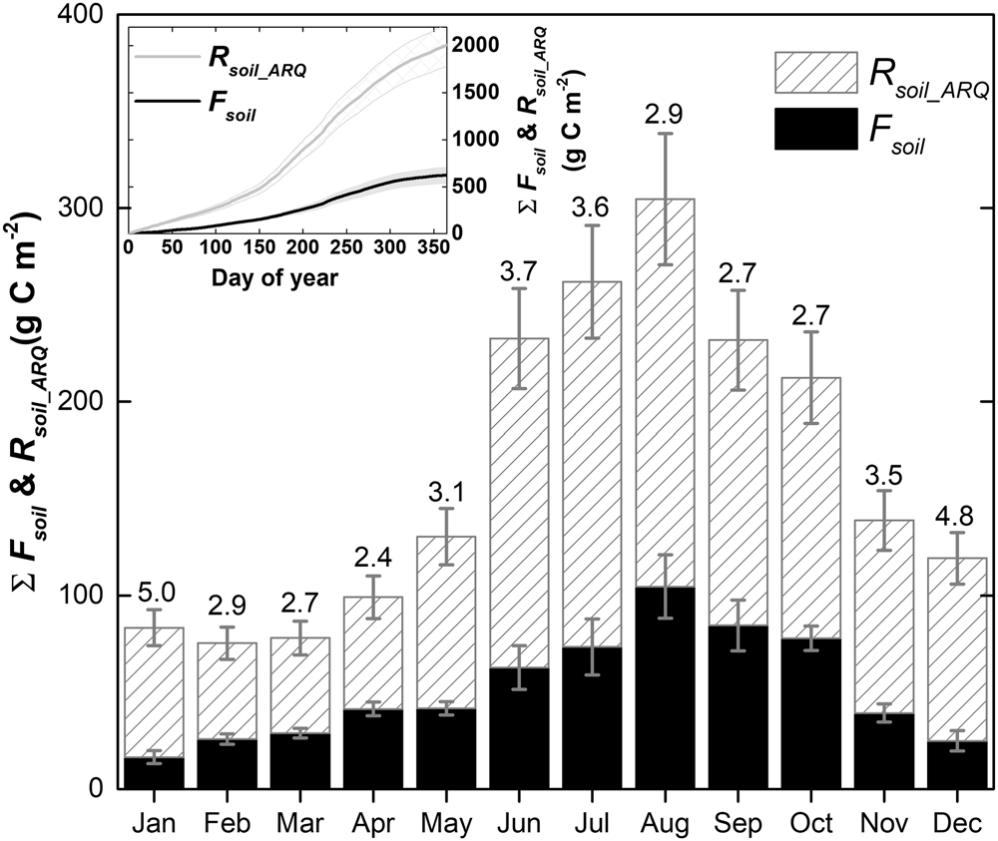
Monthly cumulative soil CO_2_ efflux (*F*_*soil*_) (with uncertainty represented as the standard error) and monthly cumulative soil CO_2_ efflux, accounting for the CO_2_ removed from the soil respiration (*R*_*soil_ARQ*_, calculated as *R*_*soil*_ multiplied by 0.9 ± 0.1/ARQ). The values above of each bar indicate the ratio *R*_*soil_ARQ*_/*F*_*soil*_. The inset figure shows the annual cumulative of *F*_*soil*_ and *R*_*soil _ARQ*_ and its uncertainty.

The annual cumulative *F*_*soil*_, including consideration of the CO_2_ loss (*R*_*soil_ARQ*_, 2012 ± 223 gC m^−2^) was 3.2 times higher than traditional estimates of *F*_*soil*_ derived using the gradient method (622 ± 86 gC m^−2^, using eq. 1). This suggests that *ca*. 1400 gC m^−2^ were removed from *R*_*soil*_ (Fig. 4) prior to efflux from the soil surface. These *ca*. 1400 gC m^−2^ represent the soil CO_2_ efflux not emitted to the atmosphere (*R*_*loss*_) in the vicinity of production. If *R*_*soil*_ was fully emitted to the atmosphere locally, by upward gaseous diffusion processes, with zero *R*_*loss*_, then *R*_*soil*_ would accurately reflect *F*_*soil*_. However, this was not the case. The smallest differences between *F*_*soil*_ using the traditional assumption of equalling *R*_*soil*_ verses using *R*_*soil_ARQ*_ were in March, April, September and October, but even then, our recalculated *F*_*soil*_ was still 2.7–3.0 times higher (Fig. 4). Maximum differences were produced in January and December, when our recalculated *F*_*soil*_ was 5.3–5.6 times higher. Our two estimates of *F*_*soil*_ (with and without accounting for *R*_*soil_ARQ*_) followed similar monthly patterns despite the differences found in ARQ. The degree of agreement between *F*_*soil*_, estimated using the gradient method (Fig. 2g), and periodic chamber measurements of *F*_*soil*_ can be found as *Supplementary Information* (Fig. 2S).

## Discussion

Given the significant role of soil carbon dynamics in determining other bio-hydro-geochemical processes in the critical zone, there is a need to better understand the dynamic nature of CO_2_ production and loss from an ecosystem. The low ARQ values we found here (ARQ ≈ 0.3, Fig. 2 and Table 1S) in comparison to oxidative ratios expected for natural organic matter (i.e., moles of O_2_ consumed per mole of CO_2_ produced during respiration of organic matter, which average ca. 1.1^24^ equivalent to ARQ = 0.9), highlight the important role of subsurface biological and non-biological processes in removing CO_2_ from *R*_*soil*_. These processes are discussed further below.

If all *R*_*soil*_ were emitted directly to the atmosphere by gaseous diffusion processes (that is, if *F*_*soil*_ = *R*_*soil*_), as is commonly assumed, *F*_*soil*_ would be on average approximately three times higher (due to ratio between ARQ theoretical/ARQ measured, 0.9/0.3). Therefore, assuming that all O_2_ consumption is associated with respiration, in this semiarid forest only 1/3 of *R*_*soil*_ is emitted directly to the atmosphere and 2/3 are removed by subsurface processes. These results are actually quite similar to those found in the only other paper that has calculated *in situ* ARQ for estimates of *F*_*soil*_^22^, which reported a mean ARQ of 0.26 and, therefore, an *R*_*soil*_ that is 3.8 times higher than *F*_*soil*_ estimated in their experimental site (Yatir forest). In that study, researchers collected CO_2_ and O_2_ samples in a pine forest overlying chalk and limestone bedrock with a mean annual precipitation of 280 mm. Despite their site receiving only 1/3 of the precipitation of our site, and therefore less potential for CO_2_ reaction with soil water, a similar ARQ was obtained. This could be attributed to a different composition (and hence oxidative ratio) of the soil organic matter undergoing decomposition, and the effect of CO_2_-consuming calcium carbonate dissolution reactions in their soils. Here, we used ARQ = 0.9 as a representative respiratory quotient (RQ) value since it is the mean value corresponding to biomolecular components of natural organic matter^24^, but if we had used for example the 0.74 value measured for a grassland soil^25^, the calculated annual *F*_*soil*_ would be 1023 g C m^−2^, which would be only 1.6 times higher *F*_*soil*_ (assuming that all *R*_*soil*_ is emitted by diffusion processes). This highlights the fact that the contribution of *R*_*soil_ARQ*_ to *F*_*soil*_ will depend on the oxidative ratio of the organic matter undergoing degradation, which could potentially change seasonally or with location. Nonetheless, our results are in accordance with Angert *et al*.^22^ and underscore the important contribution of subsurface processes in removing CO_2_ (or O_2_) from the soil gas phase prior to its efflux from the soil surface, and the need for a better understanding of the mechanisms involved in those losses.

Prior measurements of RQ have been mostly limited to laboratory experiments using air samples from natural soils or incubated soils, and we do not know of any other studies with *in-situ* and continuous estimates of RQ as a function of soil depth. In our case, assuming that only *R*_*soil*_ and diffusion of O_2_ and CO_2_ give rise to ARQ, ARQ will be equal to RQ and the oxidative ratio (OR) of organic matter undergoing degradation. In this study, the annual mean RQ (calculated as ARQ/0.76) across all depths was 0.38, which was lower than RQ values for some soils ranging from 0.82 to 1^22,26–29^, but similar to or exceeding those of other soils ranging from 0.21 to 0.40^22,30–33^. Incubation studies have found a decrease in RQ values with time, often attributed to a depletion of labile organic matter (organic acids and carbohydrates). In such conditions, the microbiota shift to metabolizing less energetically favourable compounds with lower RQ values, such as lipids, lignin and protein^34^. Therefore, the low RQ values found here might suggest that the carbon in the organic matter undergoing degradation was of relatively low oxidation state. However, RQ values were far lower than the common values of 0.88 for lignin and 0.73 for lipids^35^, suggesting that low RQ substrates cannot alone explain our results; there must also be CO_2_ or O_2_ consuming processes contributing to these very low values.

Significant soil CO_2_ losses can also be driven by DIC drainage and chemical reactions in the soil. The solubility of CO_2_ in water is described by Henry’s law, which states that the number of moles of dissolved CO_2_ plus carbonic acid per liter of water (collectively referred to as [H_2_CO_3_*]) are directly proportional to the CO_2_ partial pressure and inversely proportional to temperature. In this study, based on aqueous geochemical calculations^36^, the potential CO_2_ removed as DIC during the whole year would be 15.35 gC m^−2^. This would represent roughly 2.5% of the cumulative *F*_*soil*_ (622 gC m^−2^) and a 1.1% in the C removed from the cumulative *R*_*loss*_ (1390 gC m^−2^). These low values of downward DIC transport to groundwater are consistent with the low values of flux estimated globally^37^. Since they only had individual measurements taken at specific time points, Angert *et al*.^22^ posited that measurements and considerations of ARQ might become less important on annual and longer timescales when the effects of CO_2_ storage and release might be cancelled out. However, using continuous sensing of gas phase composition, we find the opposite. Based on our estimates, when accumulated over an annual time scale, the amount of loss was significant. This may be due, in part, to the complex topography at our mountain site, where the CO_2_-enriched water percolates to depth and is then transported laterally to groundwater discharge locations, where it may subsequently degas to the atmosphere directly^23,38,39^. Indeed, we have observed that the ephemeral stream draining the mountain study site, which runs during snowmelt or intense rainfall events, is in equilibrium with partial pressures of CO_2_ that are, on average, 5.4 ± 3.1 times higher than atmospheric^40^. Furthermore, stream discharge of highest [H_2_CO_3_*] waters is followed a couple of weeks later by a pulse of dissolved silicon derived from rock weathering^40^. With respect to chemical reactions, only those that consume CO_2_ or O_2_ lead a decrease in RQ. Potential CO_2_ consuming reactions include those wherein CO_2_ is a reactant in mineral dissolution, such as the dissolution of primary and secondary silicates^41^, (oxyhydr)oxides or calcite.

Given that plagioclase is a kinetically labile primary silicate mineral present in the soil profiles of our study site, it is reasonable to expect that some portion of the respired CO_2_ is consumed in its weathering to form kaolinite, also observed in our profiles (Table 1). The CO_2_-driven weathering of plagioclase to kaolinite consumes two moles of CO_2_ per mole of plagioclase. Numerous prior laboratory and field studies have measured rates of plagioclase dissolution at pH values similar to those of the pore waters at our site (ca. pH 5.4). Laboratory-derived weathering rates of plagioclase are typically two to three orders of magnitude higher than those derived from field data (White & Buss, 2014). Hence, whereas steady state laboratory rates are approximately 1.5 × 10^−12^ moles m^−2^ s^−1^, field-measured rates are closer 1 × 10^−14^ moles m^−2^ s^−1^ or lower (normalization in this case is to plagioclase surface area)^42^. Given the mass fraction of plagioclase in the study soils, a soil bulk density of 1.5 g cm^−3^, and assuming a specific surface area for the plagioclase as 5.6 m^2^ g^−1^ (estimated as 3/(particle density x particle radius))^43^, we calculate that the steady state rates of plagioclase dissolution could account for consumption of *ca*. 3.0 to 230 gC m^−2^ y^−1^. Importantly, plagioclase is only one of the primary silicates present in our soils; other labile silicates, such as K-feldspar and mica, will consume comparable quantities of CO_2_ during dissolution and both are present at higher mass concentrations. Nonetheless, it seems clear that silicate dissolution alone is unlikely to explain all of the CO_2_ removed in our study.

**Table 1:** Soil physicochemical characteristics and mineralogical composition.

Depth (cm)	Quartz	Plag-Feldspar	K-Feldspar	Iron Oxides	Mica	2:1 Clay	1:1 Clay	Others
0–20	44.0	7.1	8.2	1.0	13.1	17.1	4.7	3.0
20–40	46.2	5.8	6.9	0.5	14.2	18.5	5.9	2.3
40–80	40.1	4.8	6.1	0.7	13.3	16.3	5.9	4.6
Depth (cm)	Clay (%)	Silt (%)	Sand (%)	pH	Ec (µS/cm)	LOI (%)		
0–20	19.8	48.8	31.4	5.7	196.1	21.2		
20–40	27.5	44.0	28.5	5.3	199.1	5.8		
40–80	31.8	31.4	36.8	5.1	122.1	5.0		

O_2_ consuming reactions include the oxidation of Fe(II), NH_4_^+^, NO_2_^−^, mineral sulfides, H_2_S and SO_2_^44^. The rates of pyrite (FeS_2_) oxidation in regolith are controlled by the delivery of O_2_ to the weathering zone, which consumes 3.75 moles of O_2_ per mole of pyrite oxidized, and hence this can be a significant sink for O_2_ in soil systems^45^. In our site, this potential contribution may be limited (though not negligible) because of low pyrite content in the schist-derived mineral assemblage. However, biotite (mica) content in our micaceous schist derived soil is significant, representing up to 14% of the bulk soil mineral mass (Table 1), and it can contain up to three moles of Fe(II) per mole of formula, with 0.25 moles of O_2_ being consumed per mole of Fe(II) oxidized to Fe(III) during biotite weathering. Although nitrification processes were already considered in the RQ values previously shown, the deposition of calcareous atmospheric dust along with high inputs of Ca^2+^, Mg^2+^, K^+^, Na^+^, as found in the region^46^, could have contributed to lowering RQ values due to chemical reactions. Calcite dissolution plays an important role in producing and consuming CO_2_ in carbonate-containing soils^19^, with one mole of CO_2_ consumed per mole of calcite dissolved. The relative contribution of this reaction to subsurface CO_2_ consumption is unclear because CaCO_3_ does not accumulate to levels quantifiable by X-ray diffraction and soil pH (5.4) is moderately acidic. Nonetheless, the mineralogical and geochemical composition of the soil (Table 1) indicate that all of the previously mentioned reactions could consume CO_2_ and O_2_ to varying degrees, contributing the low ARQ value we measured here.

Microbial composition likely also impacts the ARQ observed in a given soil. The moles of CO_2_ produced per mole of O_2_ consumed depends, in part, on the microbial carbon-use efficiency (i.e., the ratio of growth to carbon uptake) of the heterotrophic community^47^. Hence, microbial community composition and environmental conditions (e.g. temperature, tends to decline carbon-use efficiency with increasing temperature) will likewise influence the moles of CO_2_ produced per mole of O_2_ consumed for a given substrate. The minimum ARQ was obtained at 60 cm during the snowmelt period (Fig. 2f) induced by the minimum O_2_ values. However, the maximum ARQ occurred in April. We speculate that this may be the result of the accumulation, over winter, of labile and energetically favourable organic compounds (organic acids and carbohydrates) that are oxidized by a heterotrophic microbial community activated by increasing spring temperatures. Oxidation of such compounds, containing carbon in a higher oxidation state, results in a higher ratio of moles of CO_2_ produced per mole of O_2_ consumed. Furthermore, chemolithoautotrophic and photoautotrophic organisms can assimilate CO_2_ without O_2_ production using different metabolic pathways. Photoautotrophic and chemoautotrophic organisms that fix CO_2_ and transform it into microbial biomass have been found to be highly abundant in forests^48^, with a global rate for microbial synthesis of organic C of 4.9 to 37.5 gC m^−2^ year^−1^ in different soils^49^. Methanogenic bacteria that metabolize CO_2_ to decompose organic matter to CH_4_ under anaerobic conditions^50^ have been observed even in well aerated soils such as those found in deserts^51^. Therefore, the low ARQ and RQ values found in our soils could indicate one or several processes whereby (i) CO_2_ is being removed laterally as dissolved H_2_CO_3_*, (ii) CO_2_ and O_2_ are consumed in geochemical reactions, or (iii) a biological O_2_ consumption occurs without emission of CO_2_ and *vice versa*.

Subsurface CO_2_ consumption has been studied both in soil-atmosphere CO_2_ exchanges and in CO_2_ exchanges at the ecosystem level. Roland *et al*.^52^ used a chemical carbonate weathering model to explain non-biological fluxes detected at ecosystem scale in a karst, finding that the CO_2_ coming from deeper layers at night could be stimulating carbonate dissolution and, thus, consuming CO_2_. Hamerlynck, *et al*.,^53^ found a negative *F*_*soil*_ at night in a Chihuahuan desert shrubland, both using an automatic soil chamber and using the gradient method with CO_2_ sensors buried in the shallowest layer, similarly attributing the CO_2_ consumption to carbonate dissolution. Additionally, temperature influences on the solubility of CO_2_ (Henry’s Law) were suggested in explaining negative *F*_*soil*_ in Antarctic dry valley ecosystems^54,55^, and soil electrical conductivity and pH were correlated with CO_2_ uptake in alkaline desert soils^56^. All of these studies found negative *F*_*soil*_, highlighting that CO_2_ consumptive processes in the soil were higher than CO_2_ production processes. This is not unexpected in such ecosystems, where *R*_*soil*_ is very low due to low biological activity and therefore even small changes in *R*_*soil*_ can change the sign of the soil-atmosphere CO_2_ gradient. In our ecosystem, *F*_*soil*_ was always positive, but the complementary O_2_ measurements provided a novel insight, confirming that even in ecosystems with high biological production, non-biological processes are masked by high *R*_*soil*_ and therefore, are difficult to detect from *F*_*soil*_ measurements alone.

In conclusion, this study highlights the important and dynamic, but often overlooked, roles played by subsurface transport and weathering processes that differentiate *R*_*soil*_ from surface measures or estimates of *F*_*soil*_. As Angert *et al*.^22^ noted, variations in the ARQ in acidic and neutral soils (as we have here) are likely tied to substrates and processes not well understood at present, and such processes warrant further research. Therefore, we must change our point of view regarding *R*_*soil*_ studies from an inappropriately conceived system in which all CO_2_ is produced by biology, to a dynamic system where the soil CO_2_ is produced and removed by the interaction of combinatorial biological processes, hydrologic transport, and associated geochemical reactions. Because the fraction of *R*_*soil*_ contributing to *F*_*soil*_ depends on the ARQ chosen, we recommend that future *F*_*soil*_ studies use a combination of soil CO_2_ and O_2_ sensors to determine ARQ values. Such an approach can yield important information to quantify the CO_2_ removed by biological and non-biological processes. ARQ and RQ values are key in estimating CO_2_ sinks deduced from changes in atmospheric O_2_ concentration^57^ and are highly influential in evaluating ecosystem productivity. Currently, ecosystem productivity is estimated using values of net ecosystem exchange, as the sum of gross primary production (GPP) and ecosystem respiration (*R*_*eco*_). This may be problematic because that *R*_*eco*_ consists of an aboveground component attributed to plant respiration and a belowground component, *F*_*soil*_ that we now know may incompletely quantify soil respiration. In our ecosystem, if soil CO_2_ losses were calculated from *F*_*soil*_ alone, GPP estimates would be erroneously low, and if this is consistent across other ecosystems, it could have enormous implications on carbon exchange studies from ecosystem to global scale.

## Material and Methods

### Site description

The field site is a mixed conifer forest located at 2573 m a.s.l. on Mt. Bigelow north of Tucson, Arizona, in the Santa Catalina Mountains-Jemez River Basin Critical Zone Observatory^58^. The climate is semi-arid, with a mean annual temperature of 9.4 °C and mean annual precipitation of 750 mm, falling mostly during the summer monsoon. Snow falls during winter, usually persisting from December to March. Ponderosa pine (*Pinus ponderosa*) and Douglas fir (*Pseudotsuga menziesii*) dominate the site with a mean canopy height of 10 m. The soil has a sandy loam texture of 32.3% sand, 41.4% silt and 26.4% clay with a pH of 5.4 and a depth to bedrock of *ca*. 1 m. Additional information about mineral composition and other soil proprieties can be found in Table 1.

### Experimental design

Field measurements were conducted during the complete calendar year of 2015. Three instrumented pedons were equipped to measure each of the following, using co-located sensors: temperature and humidity (5 TM, Decagon, USA), O_2_ molar fraction (SO-110, Apogee, USA; Manufacturer reports a sensitivity of 26 µV per 0.01% and repeatability < 0.1% of reading), and CO_2_ molar fraction at 10, 30 and 60 cm depth. A drift correction was applied to the O_2_ sensors assuming a constant linear signal decrease as the manufacturer reported (1 mV per year). The measurement range of the CO_2_ sensors was up to 10,000 ppm at 10 cm and 20,000 ppm at 30 and 60 cm (GMM222 and GMM221, Vaisala, Finland; accuracy 1.5%, repeatability 2% of reading). Both CO_2_ and O_2_ values were corrected for variations in temperature, humidity, and pressure per instructions from the manufacturer. Atmospheric pressure, air temperature, and precipitation were obtained from a meteorological tower. Data-loggers (CR1000, Campbell scientific, USA) collected measurements every 30 s and stored 30 min averages. The instrumented pedons are separated from each other by distances of ca. 10 meters, and they are located, respectively, on a south facing slope, a north facing slope, and in a convergent valley position within a zero order basin. One-way ANOVA for mean values of soil temperature, soil water content, CO_2_ and O_2_ between 3 pedons at 3 depths, showed significant differences among all the means at each depth for each variable. Here, we aggregated the three pedons and analysed the average values and their standard error to show the uncertainty in the spatial variability.

### Procedure to estimate *F*_*soil*_

Estimates of *F*_*soil*_ were obtained using the gradient method through the equation^59^:

1 $${F}_{soil}=-\,\rho {k}_{s}\frac{\partial c}{\partial x}$$

where *F*_*soil*_ (µmol CO_2_ m^−2^ s^−1^), *ρ* is the air density (mol air m^−3^), $\partial c$ is the CO_2_ molar fraction gradient (µmol CO_2_ mol air^−1^) calculated using the difference between atmospheric CO_2_ molar fraction (400 ppm) and the CO_2_ value at 10 cm depth, $\partial x$ is the vertical gradient (m) and *k*_*s*_ is the *in situ* CO_2_ transfer coefficient (m^2^ s^−1^) obtained by rearranging Eq. 1:

2 $${k}_{s}=-\,\frac{{F}_{chamber}\,\partial x}{\rho \,\partial c}$$

where *F*_*chamber*_ was measured by a portable soil CO_2_ efflux chamber (Li-8100, Li-Cor, USA) from 18 collars around the instrumented pedons, follow a transect from the south face to the north face going through the valley, every two weeks during the months without snow cover (n=20). Later, *k*_*s*_ was modelled using a power function (*k*_*s*_*/ D*_*a*_ = a *θ*_*a*_^b^) of the soil air porosity (*θ*_*a*_ = soil porosity-soil water content), where *D*_*a*_ is the diffusion coefficient of CO_2_ in free air (m^2^ s^−1^) and *a* and *b* are coefficients obtained by least squares regression.

### Procedure to estimate ARQ

The ratio of soil CO_2_ efflux to soil O_2_ influx, designated as apparent respiratory quotient (ARQ), was estimated following Angert *et al*.^22^:

3 $$ARQ=\frac{{F}_{C{O}_{2}}}{{F}_{{O}_{2}}}=\frac{-\rho {D}_{{S}_{CO2}}\,\frac{\partial c}{\partial z}}{-\rho {D}_{{S}_{O2}}\frac{\partial o}{\partial z}}$$

simplifying,

4 $$ARQ=\frac{-{D}_{{S}_{CO2}}\,}{-{D}_{{S}_{O2}}}\frac{\partial c}{\partial o}=-\,0.76\frac{\partial c}{\partial o}$$

where the constant “0.76” is derived from the ratio of CO_2_/O_2_ diffusion coefficients in air, $\partial \,$c is the CO_2_ molar fraction gradient calculated using the discrete difference between the atmosphere and the CO_2_ value at each depth and $\partial \,$o is the O_2_ molar fraction gradient calculated using the difference between atmosphere and the O_2_ value at each depth. Consumption of either soil CO_2_ or soil O_2_ will decrease the ARQ; consumption of soil CO_2_ decreases the difference in the numerator ($\partial c$) and hence decreases ARQ, whereas consumption of soil O_2_ increases the difference represented in the denominator ($\partial o$), and hence also decreases ARQ.

ARQ values have previously only been reported by Angert *et al*.^22^, who found that ARQ ranged from 0.14–1.23 across six different experimental sites. Most previous studies have focused either on the *respiratory quotient* (RQ), defined as the moles of CO_2_ produced per mole of O_2_ consumed during *R*_*soil*_, or the *oxidative ratio* (OR), defined as moles of O_2_ consumed per mole CO_2_ produced (i.e., 1/RQ). Therefore, if we assume that only *R*_*soil*_ drives ARQ, it will be equal to RQ or 1/OR.

The natural biochemical variation in RQ is large depending on the kind of compound undergoing oxidation, ranging from (mean values reported for each biomolecular type) 1.47 for organic acids, 1.00 for carbohydrates, 0.95 for soluble phenolics, 0.88 for proteins and lignins, and 0.73 for lipids (OR values in Randerson *et al*.,^35^). From stoichiometric considerations, mean RQ values were calculated as 0.95 for different types of wood and 0.89 for humic acid and humin (OR values in Severinghaus^28^). In soils, RQ values have been reported to vary from 0.83–0.95 for different biomes inside Biosphere 2^28^, 0.82–1.04 for Boreal, Temperate Subtropical and Mediterranean ecosystems^29^, 0.90 in a cool temperate deciduous forest^27^, and a mean value of 1 in the Amazonian tropical forest^26^. Therefore, based on previous research, an ARQ value of *ca*. 0.9 ± 0.1 is consistent with *R*_*soil*_ and diffusion processes alone. However, ARQ values below this would indicate removal of CO_2_ or O_2_ by non-respiratory processes^22^. Therefore, assuming both abiotic O_2_ removal and autotrophic microorganisms in the soil are negligible, to estimate the *F*_*soil*_ taking into account the CO_2_ loss from the soil, one can multiply *R*_*soil*_ (or *F*_*soil*_, assuming that all *R*_*soil*_ is emitted to the atmosphere by gaseous diffusion processes, and therefore, *F*_*soil*_ = *R*_*soil*_) by 0.9 ± 0.1 /ARQ, as was done in the current study and previously by Angert *et al*.^22^.

## Electronic supplementary material

Supplementary Information
