# Supplementary material for: A considerable fraction of soil-respired CO2 is not emitted directly to the atmosphere
Source: Sci Rep. 2018 Sep 10;8:13518. doi: 10.1038/s41598-018-29803-x (PMC6131168; doi:10.1038/s41598-018-29803-x)
Supplement: Supplementary file 1 — Supplementary Information [file 41598_2018_29803_MOESM1_ESM.docx]

Supplementary Information

A considerable fraction of soil-respired CO_2_ is not emitted directly to the atmosphere.

**Enrique P. Sánchez-Cañete^1,2,*^, Greg A. Barron-Gafford^1,3^, and Jon Chorover^4^**

^1^ B2 Earthscience, Biosphere 2, University of Arizona, Tucson, 85721, USA

^2^ Departamento de Física Aplicada, Universidad de Granada, Granada, 18071, Spain

^3^ School of Geography and Development, University of Arizona, Tucson, 85721, USA

^4^ Department of Soil, Water and Environmental Science, University of Arizona, Tucson, 85721, USA

*corresponding author: enripsc@ugr.es

|  | Mean | SD | Min | Max |
| --- | --- | --- | --- | --- |
| Atmospheric Pressure (kPa) | 74.46 | 0.42 | 73.22 | 75.22 |
| Air T (^o^C) | 9.42 | 6.63 | -10.22 | 21.94 |
| Soil T 10cm (^o^C) | 9.14 | 5.05 | 0.88 | 18.00 |
| Soil T 30cm (^o^C) | 9.35 | 4.34 | 2.68 | 16.35 |
| Soil T 60cm (^o^C) | 9.43 | 3.95 | 3.78 | 15.62 |
| VWC 10cm (%) | 0.20 | 0.03 | 0.13 | 0.28 |
| VWC 30cm (%) | 0.19 | 0.03 | 0.13 | 0.34 |
| VWC 60cm (%) | 0.19 | 0.03 | 0.15 | 0.29 |
| χ_CO2_ 10cm (ppm) | 2537 | 1308 | 1026 | 8987 |
| χ_CO2_ 30cm (ppm) | 5693 | 2368 | 2325 | 13413 |
| χ_CO2_ 60cm (ppm) | 6407 | 2650 | 2123 | 14028 |
| O_2_ 10cm (%) | 20.27 | 0.31 | 18.63 | 20.67 |
| O_2_ 30cm (%) | 19.27 | 0.39 | 17.32 | 20.00 |
| O_2_ 60cm (%) | 18.04 | 2.48 | 5.75 | 19.93 |
| ARQ 10cm | 0.28 | 0.07 | 0.08 | 0.54 |
| ARQ 30cm | 0.33 | 0.05 | 0.12 | 0.48 |
| ARQ 60cm | 0.25 | 0.09 | 0.02 | 0.45 |
| F_soil_ | 1.64 | 0.90 | 0.15 | 4.96 |

**Table 1S**. Average, standard deviation, minimum and maximum for each variable during 2015.

|  | T10 | T30 | T60 | VWC10 | VWC30 | VWC60 | χ_CO2_10 | χ_CO2_30 | χ_CO2_60 | O_2_10 | O_2_30 | O_2_60 | ARQ10 | ARQ30 | ARQ60 | *F_soil_* |
| --- | --- | --- | --- | --- | --- | --- | --- | --- | --- | --- | --- | --- | --- | --- | --- | --- |
| T10 |  | **0.99** | **0.97** | -0.53 | -0.58 | -0.51 | 0.57 | **0.75** | **0.86** | -0.39 | -0.67 | 0.21 | 0.32 | 0.23 | 0.69 | **0.86** |
| T30 | **0.99** |  | **1.00** | -0.47 | -0.55 | -0.48 | 0.63 | **0.79** | **0.90** | -0.47 | **-0.70** | 0.21 | 0.28 | 0.25 | 0.67 | **0.89** |
| T60 | **0.97** | **1.00** |  | -0.42 | -0.53 | -0.45 | 0.67 | **0.82** | **0.92** | -0.52 | **-0.72** | 0.21 | 0.26 | 0.27 | 0.65 | **0.91** |
| VWC10 | -0.53 | -0.47 | -0.42 |  | **0.81** | **0.75** | 0.27 | 0.07 | -0.15 | -0.45 | -0.07 | -0.38 | -0.21 | 0.11 | -0.65 | -0.19 |
| VWC 30 | -0.58 | -0.55 | -0.53 | **0.81** |  | **0.90** | 0.04 | -0.15 | -0.33 | -0.20 | 0.04 | -0.53 | -0.16 | 0.00 | **-0.72** | -0.34 |
| VWC 60 | -0.51 | -0.48 | -0.45 | **0.75** | **0.90** |  | 0.06 | -0.12 | -0.28 | -0.23 | 0.05 | -0.66 | -0.20 | 0.06 | **-0.76** | -0.29 |
| χ_CO2_10 | 0.57 | 0.63 | 0.67 | 0.27 | 0.04 | 0.06 |  | **0.94** | **0.84** | **-0.94** | **-0.91** | -0.10 | 0.30 | 0.27 | 0.16 | **0.85** |
| χ_CO2_30 | **0.75** | **0.79** | **0.82** | 0.07 | -0.15 | -0.12 | **0.94** |  | **0.94** | **-0.82** | **-0.89** | -0.02 | 0.33 | 0.38 | 0.34 | **0.93** |
| χ_CO2_60 | **0.86** | **0.90** | **0.92** | -0.15 | -0.33 | -0.28 | **0.84** | **0.94** |  | **-0.70** | **-0.82** | 0.11 | 0.27 | 0.31 | 0.55 | **0.93** |
| O_2_10 | -0.39 | -0.47 | -0.52 | -0.45 | -0.20 | -0.23 | **-0.94** | **-0.82** | **-0.70** |  | **0.85** | 0.14 | -0.04 | -0.15 | 0.00 | **-0.70** |
| O_2_30 | -0.67 | **-0.70** | **-0.72** | -0.07 | 0.04 | 0.05 | **-0.91** | **-0.89** | **-0.82** | **0.85** |  | 0.09 | -0.34 | -0.12 | -0.22 | **-0.84** |
| O_2_60 | 0.21 | 0.21 | 0.21 | -0.38 | -0.53 | -0.66 | -0.10 | -0.02 | 0.11 | 0.14 | 0.09 |  | -0.01 | -0.21 | 0.67 | 0.10 |
| ARQ10 | 0.32 | 0.28 | 0.26 | -0.21 | -0.16 | -0.20 | 0.30 | 0.33 | 0.27 | -0.04 | -0.34 | -0.01 |  | 0.19 | 0.17 | 0.36 |
| ARQ30 | 0.23 | 0.25 | 0.27 | 0.11 | 0.00 | 0.06 | 0.27 | 0.38 | 0.31 | -0.15 | -0.12 | -0.21 | 0.19 |  | 0.07 | 0.29 |
| ARQ60 | 0.69 | 0.67 | 0.65 | -0.65 | **-0.72** | **-0.76** | 0.16 | 0.34 | 0.55 | 0.00 | -0.22 | 0.67 | 0.17 | 0.07 |  | 0.48 |
| *F_soil_* | **0.86** | **0.89** | **0.91** | -0.19 | -0.34 | -0.29 | **0.85** | **0.93** | **0.93** | **-0.70** | **-0.84** | 0.10 | 0.36 | 0.29 | 0.48 |  |

**Table 2S**. Correlation matrix (R^2^) of daily values during 2015, bold values show R^2^>0.7.





**Figure 1S**. Box-and-whisker plot of edaphic variables and apparent respiratory quotient (ARQ) at half-hour scale in three instrumented soil pedons, grouped by month during 2015. Each box corresponds to the inter-quartile range. Mean is the horizontal line in the box. Whiskers extend from percentiles 5 to 95 and “x” correspond to percentiles 1 and 99.

Monthly descriptive statistics for edaphic variables and ARQ in the three instrumented soil pedons at half-hour scale can be found in the box-and-whisker plot (Figure 1S). At all depths, the months of December-January were the coldest and July-August the warmest. SWC was lowest in June and highest in January. CO_2_ concentrations were lowest in April at 10 cm, but in January at 30 cm and 60 cm; the highest CO_2_ values occurred in August at all depths. At 10, 30, and 60 cm the highest/lowest O_2_ values were in April/August, January/August, and April/February, respectively. The highest-lowest ARQ values were in April-January, November-May and June-February at 10 cm, 30 cm, and 60 cm, respectively. Similar annual patterns were found in T, SWC, CO_2_, and O_2_ at their respectively depths. However, the ARQ annual pattern was different at each depth. ARQ followed a bimodal pattern at 10 cm, with a maximum in April coinciding with the maximum O_2_ values and minimum CO_2_ values. At 30 cm, ARQ exhibited high variation around its mean value and did not show a clear annual pattern, but ARQ at 60 cm followed a clear monomodal annual pattern, inversely related to SWC.


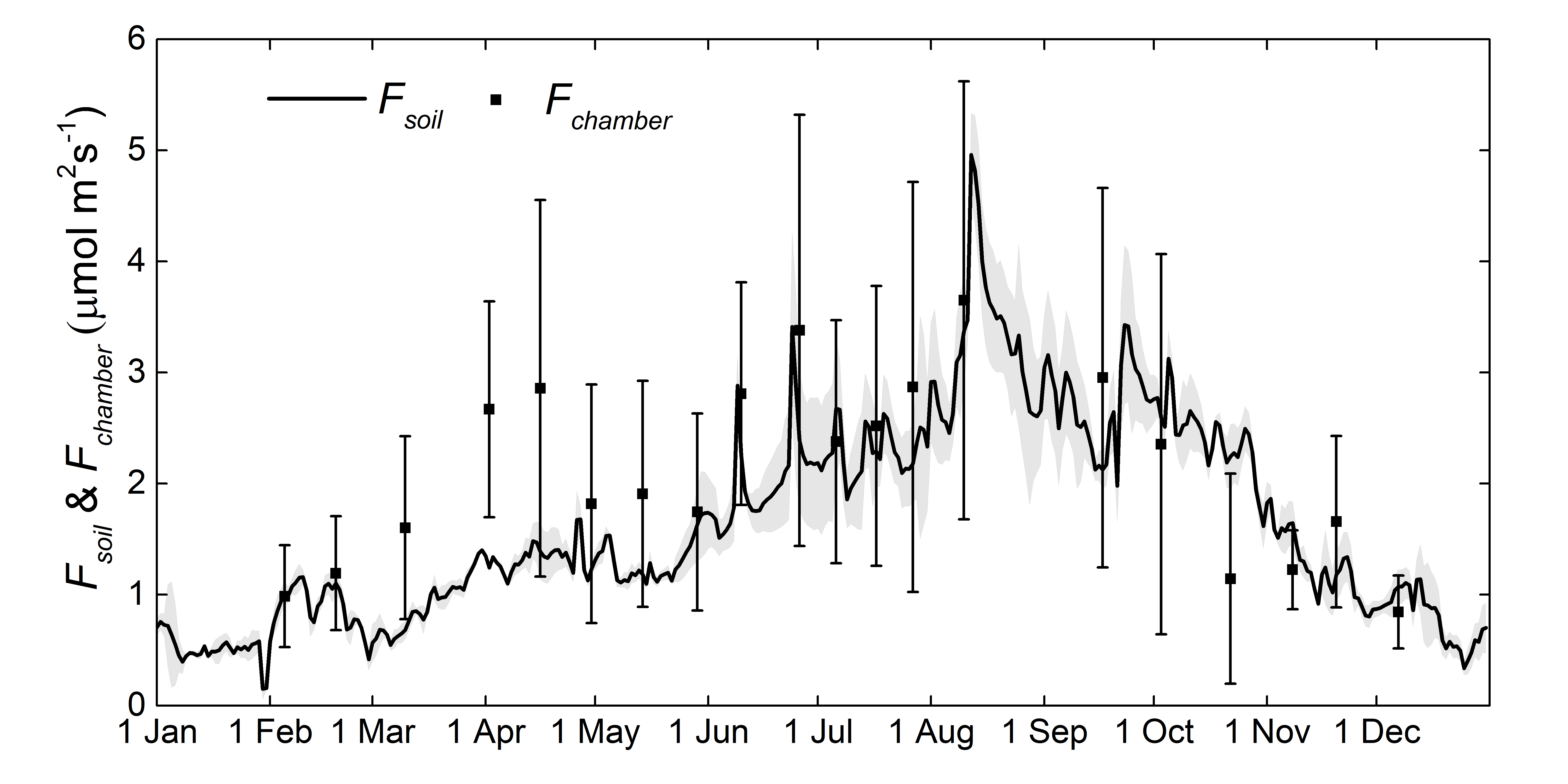


**Figure 2S**. Soil CO_2_ efflux and standard error obtained using the gradient method (*F_soil_*) and Soil CO_2_ efflux and standard deviation obtained using a manual chamber Li-Cor 8100 (*F*_chamber_) during 20 campaigns in 2015.
